# The characteristics of pain and dysesthesia in patients with diabetic polyneuropathy

**DOI:** 10.1371/journal.pone.0263831

**Published:** 2022-02-17

**Authors:** Sandra Sif Gylfadottir, Mustapha Itani, Alexander Gramm Kristensen, Pall Karlsson, Thomas Krøigård, David L. Bennett, Hatice Tankisi, Niels Trolle Andersen, Troels Staehelin Jensen, Søren Hein Sindrup, Nanna Brix Finnerup

**Affiliations:** 1 Danish Pain Research Center, Department of Clinical Medicine, Aarhus University, Aarhus, Denmark; 2 Department of Neurology, Aarhus University Hospital, Aarhus, Denmark; 3 Department of Neurology, Odense University Hospital, Odense, Denmark; 4 Department of Clinical Neurophysiology, Aarhus University Hospital, Aarhus, Denmark; 5 Core Center for Molecular Morphology, Section for Stereology and Microscopy, Aarhus University, Denmark; 6 Nuffield Department of Clinical Neuroscience, University of Oxford, Oxford, United Kingdom; 7 Biostatistics, Department of Public Health, Aarhus University, Aarhus, Denmark; Weill Cornell Medicine-Qatar, QATAR

## Abstract

**Introduction/aims:**

Patients with diabetic polyneuropathy (DPN) may experience paresthesia, dysesthesia, and pain. We aimed to characterize the predictors, symptoms, somatosensory profile, neuropathy severity, and impact of painful DPN and dysesthetic DPN.

**Methods:**

This study was a cross-sectional study of type 2 diabetes patients with confirmed DPN, diagnosed using widely accepted methods including a clinical examination, skin biopsy, and nerve conduction studies.

**Findings:**

Of 126 patients with confirmed DPN, 52 had DPN without pain or dysesthesia, 21 had dysesthetic DPN, and 53 painful DPN. Patients with painful DPN were less physically active and suffered from more pain elsewhere than in the feet compared to patients with DPN without pain. Patients with painful DPN had the largest loss of small and large sensory fiber function, and there was a gradient of larger spatial distribution of sensory loss from DPN without dysesthesia/pain to dysesthetic DPN and to painful DPN. This could indicate that patients with dysesthesia had more severe neuropathy than patients without dysesthesia but less than patients with painful DPN. Patients with dysesthetic and painful DPN had higher symptom scores for depression and fatigue than those without dysesthesia/pain with no difference between dysesthetic and painful DPN.

**Conclusions:**

There was a gradient of increasing sensory loss from DPN without dysesthesia/pain to dysesthetic DPN and to painful DPN. Pain and dysesthesia are common in DPN and both interfere with daily life. It is therefore important to consider dysesthesia when diagnosing and treating patients with neuropathy.

## Introduction

Diabetic polyneuropathy (DPN) is one of the most common complications to type 2 diabetes, affecting up to 50% of the patients [1]. Of those, around 50% have painful DPN [2, 3], and with a high global prevalence of type 2 diabetes [3, 4], it represents one of the major causes of neuropathic pain.

Little is known about the risk factors for developing neuropathic pain in those with DPN [2, 3, 5]; however, studies consistently point to an increasing severity of chronic sensory polyneuropathy as a risk factor for neuropathic pain in patients with longstanding diabetes [5–8].

Patients with lesions or disease of the somatosensory nerve system may experience spontaneuous or evoked sensations ranging from paresthesia (an abnormal sensation) over dysesthesia (an unpleasant abnormal sensation, wheter spontaneous or evoked) to pain, which is defined as an unpleasant sensory and emotional experiences associated with, or resembling that associated with, actual or potential tissue damage [9, 10]. These symptoms are well-characterized in central neurological disorders. Patients may report both pain and dysesthesia, but a large proportion of patients with stroke or spinal cord injury report only dysesthesia, either spontaneous dysesthesia and/or dysesthesia evoked by e.g. cold and light touch [11–15]. Dysesthesia can be distressing whether painful or not [16] and should be considered in the assessment of patients as they may not report dysesthesia if only asked about pain. In stroke and spinal cord injury, early evoked pain or dysesthesia to light touch, cold or pinprick have been found to predict the later development of central neuropathic pain, with more patients reporting evoked dysestesia (unplesantness) than pain [17, 18], emphasizing the importance of assessing dysesthesia. While pain and sensory loss are often adressed in the context of DPN, no attention has been given to dysesthesia, and we do not know if pain and dysesthesia share important features with respect to predictors, mechanisms, and impact. We have therefore separated pain and dysesthesia in the present study.

In this study, we hypothesized that patients with dysesthesia had more severe neuropathy than patients without dysesthesia but less than patients with neuropathic pain. We also aimed to describe the predictors, symptoms, somatosensory profile and impact of dysesthetic and painful DPN.

## Materials and methods

### Study design and patients

Patients with DPN participating in a cross-sectional detailed phenotyping study of 389 patients with type 2 diabetes performed from October 2016 to October 2018 [19] were included in this study. The patients had participated in a questionnaire survey of 5,514 type 2 diabetes patients from a large prospective cohort study: the Danish Centre for Strategic Research in Type 2 diabetes (DD2) [20, 21]. Briefly, in this study we included all 126 patients with confirmed DPN diagnosed according to the Toronto Diabetic Neuropathy Expert Group criteria [22] and all patients had abnormal nerve conduction studies (NCS) or/and intra epidermal nerve fiber density (IENFD).

We excluded patients with other causes of polyneuropathy and other neurological or pain disorders that could not be distinguished from the symptoms of DPN. We also included 97 controls without diabetes, severe illness or chronic pain of similar age and sex as the patients. They were recruited by flyers and within the social circle of the patient i.e. spouses and friends. The characteristics of the control group without diabetes is described previously [19]. Data from the control group was only used for comparison in bedside sensory mapping.

### Interview and neurological examination

The patients came in for a 1-day visit that consisted of a structured interview, a clinical examination, and a self-administered questionnaire as described previously [19]. The interview focused on symptoms of neuropathy (duration, localization, and type of symptoms). Patients were also asked about pain medicine and comorbidities, including diseases that cause polyneuropathy and pain. We measured weight, height, blood pressure, and took blood samples for glycated hemoglobin (HbA1c), cholesterol, and triglycerides on the same day as the examination.

The bedside neurological examination was performed in a quiet environment and consisted of sensory mapping of lower extremities: light brush stroking (SENSELab Brush-05; Somedic AB, Hörby, Sweden), pinprick (Owen Mumford Neuropen with sterile neurotips and Semmes-Weinstein monofilament no 5.88 (bending force 75.9 g/745 mN), Stoelting, Wood Dale, IL, USA) and cold (20°C) and warm (40°C) thermal rolls (Somedic AB, Hörby, Sweden). All modalities were tested in a control area with normal sensation (upper thigh or chest) and then examined starting in the toes and moving proximally. Patients were asked if the stimulus was the same or similar to the control stimulus, less intense, more intense or painful. If it was painful patients rated the pain on the Numeric Rating Scale (NRS). We started the examination systemically on the right side. We also assessed reflexes and muscle strength. To ensure consistency between the 2 investigators, we made a detailed description of the examination, trained together, and tested regularly for agreement.

### Questionnaire

All patients filled in a questionnaire on the day of the clinical examination. The questionnaire consisted of questions about occupational status, educational level, smoking and alcohol consumption (> 7/14 units of alcohol per week (women/men), which is the maximum safe amount recommended by the Danish Health Authority). Additionally, the patients reported how often on average per week they were physically active.

The patients were asked to rate their overall quality of life (QoL) on the 0–10 NRS, where 10 indicated the best QoL possible and 0 the worst during the last 7 days [23]. We asked about sleep disturbance, symptoms of depression and anxiety as well as pain interference with daily life (e.g., with social, recreational and physical activities) using the Patient-Reported Outcome Measurement Information System (PROMIS®) 4a short form v1.0. The scores were converted into PROMIS T scores, which are standardized relative to an American/US reference population, and to categories of impairment ranging from normal through mild impairment and moderate impairment to severe impairment [24, 25].

Patients with dysesthesia and pain filled in the Neuropathic Pain Symptom Inventory (NPSI), a questionnaire designed to evaluate the symptoms of neuropathic pain, their presence, character, and intensity [26]. Although not developed or validated to assess dysesthesia, patients with dysesthesia were asked to characterize their dysesthesia the using NPSI with and the term “pain” in the questionnaire replaced with “dysesthesia”.

### Quantitative sensory testing

We used a reduced version (9 out of 13 parameters) of the standardized quantitative sensory testing (QST) protocol of the German Research Network for Neuropathic Pain (DFNS) [27, 28]. The thermotest (Somedic Horby, Sweden) was used to examine cold detection threshold (CDT), warm detection threshold (WDT), paradoxical heat sensation (PHS), thermal sensory limen (TSL), vibration detection threshold (VDT), dynamic mechanical allodynia (DMA), pinprick hyperalgesia, the short version (2/5) of mechanical pain sensitivity (MPS), and pressure pain threshold (PPT). Patients were examined on the dorsum of the right foot corresponding to the S1 segment except for VDT, which was assessed on the right medial malleolus. We used Equista, a data analysis system that transfers data into standard normal distribution (Z-scores) adjusting for age, sex, and body localization [29].

### Nerve conduction studies and intraepidermal nerve fiber density

The NCS included examination of sural nerve bilaterally and the median, peroneal, and tibial nerves unilaterally and we defined polyneuropathy as ≥ 2 nerves with ≥ 1 abnormal parameters with at least 1 sural nerve being abnormal compared with a laboratory control sample [30–32]. Intraepidermal nerve fiber density (IENFD) was considered abnormal if it was lower than the 5th centile for age- and sex-matched healthy controls [19, 33, 34].

### Neuropathy subgroups

Small fiber neuropathy (SFN), mixed fiber neuropathy (MFN) and large fiber neuropathy (LFN) were defined according to Itani et al. [35]. SFN as one of following; abnormal pinprick or thermal sensation (bedside or QST) or abnormal IENFD, LFN as one of following; bedside abnormal vibration sensation or ankle reflexes or MDT or abnormal NCS and MFN if not fulfilling criteria for SFN or LFN [35].

### Definition of DPN, painful DPN, and dysesthetic DPN

Patients were asked if they had constant or recurring pain in the feet, and those with no pain were asked if their abnormal sensory symptoms in the feet were unpleasant (dysesthesia). In patients with no pain or dysesthesia, we did not systematically differentiate between positive and negative sensory symptoms and thus did not record whether the patient had positive sensory symptoms that were not unpleasant (paresthesia). The intensity of pain was scored on an 11-point NRS, 0–10, with 0 indicating no pain and 10 worst pain imaginable. The intensity of dysesthesia/unpleasantness was scored on the NRS with 0 indicating no dysesthesia/unpleasantness and 10 worst dysesthesia/unpleasantness imaginable.

Patients with confirmed DPN according to the Toronto consensus criteria were divided into 1. Painful DPN, which included patients with neuropathic pain according to NeuPSIG grading system for neuropathic pain, i.e. patients with pain in both feet, 2. Dysesthetic DPN, which included patients with no pain but with unpleasant abnormal sensations in the feet, and 3. DPN without dysesthesia/pain, which included patients that denied any unpleasant or painful sensations in the feet. Paresthesia was included in this group [22, 36].

### Ethics statement

All participants signed an informed written consent prior to the examination and the study was approved by the Regional Research Ethics Committee of Central Denmark Region (file number 1-10-72-130-16).

### Statistical analysis

For data analysis, we used the STATA version 14 (StataCorp LLC, TX) and R Core Team (2019) [37, 38]. Data were presented as means with standard deviation or as medians with interquartile range (IQR) and categorical data as numbers with percentages. Group comparisons were done with analysis of variance (ANOVA), Kruskal-Wallis test, or Fisher’s exact test between all 3 DPN groups and when significant, we used student’s t-test, Mann-Whitney U test, or Fisher’s exact test for paired comparisons. For the calculation of bedside spreading of sensory loss and gain, the legs were divided into 5 areas (as shown in Fig 3) and we calculated the percentages of patients with abnormalities in each area and depicted the results in the figure. To provide a sum score of bedside sensory loss for each patient, we counted the number of areas with abnormalities. There was no significant difference between sum scores on the right and left leg, confirming symmetrical pattern of sensory loss. We then used Spearman’s rank order correlation to estimate the strength and direction of the association between the sum scores of bedside sensory loss and DPN without dysesthesia/pain, dysesthetic (but non-painful) DPN, and painful DPN assuming a monotonic relationship. We used logistic regressions to estimate odds ratios for having symptoms of anxiety, depression and fatigue (yes/no) for each of the three neuropathy phenotypes.

## Results

**Fig 1:**
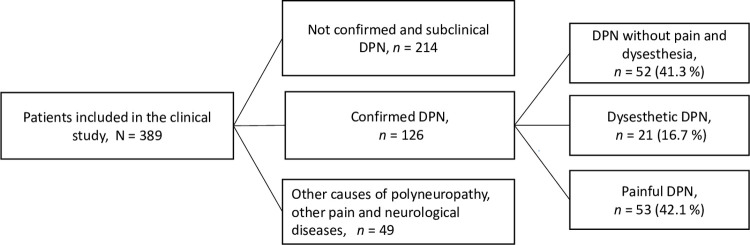
Flow diagram of patient inclusion and diagnosis of confirmed DPN according to the Toronto criteria [22] and the NeuPSIG grading of neuropathic pain [36]. For a detailed description of the recruitment, please refer to [19].

The study population consisted of 126 patients with confirmed DPN (Fig 1). Of those, 52 (41.3%) had DPN without dysesthesia or pain, 21 (16.7%) had dysesthetic DPN, and 53 (42.1%) had painful DPN. Patients found it easy to separate pain and non-painful dysesthesia (unpleasantness).

There were no differences between the three groups of patients in terms of sex, age, BMI, time since diabetes diagnosis, educational level, and occupational status (Table 1). Fewer patients with painful DPN reported physical activity ≥ 3 times per week than those with no dysesthesia/pain and dysesthesia. Patients with painful DPN reported more pain in other body sites than the feet/legs than DPN patients without dysesthesia/pain. They also used more analgesics, including both drugs normally prescribed for neuropathic pain (TCAs, gabapentin, pregabalin, and SNRIs) and non-prescription drugs (paracetamol and NSAIDs) compared to DPN patients without dysesthesia/pain (Table 1).

**Table 1:** Characteristics of patients with confirmed DPN without dysesthesia/pain, dysesthetic DPN and painful DPN.

	DPN without dysesthesia/pain	Dysesthetic DPN	Painful DPN	*P*
N = 126 (100%), in row	52 (41.3)	21 (16.7)	53 (42.1)	
***Demographics***				
Sex, female, n (%)	14 (26.9)	6 (28.6)	21 (39.6)	0.39
Age, years, median (IQR)	70.4 (62.0; 73.0)	62.2 (52.4; 71.6)	66.3 (57.5; 70.2)	0.078
BMI (kg/m^2^), median (IQR)	31.4 (28.7; 35.6)	35.1 (28.9; 38.8)	34.0 (29.5; 38.5)	0.095
Time since diabetes diagnosis, years, median (IQR)	6.1 (4.4;7.1)	6.4 (3.5; 7.1)	6.1 (4.7; 7.7)	0.75
Alcohol consumptions > 7/14 units per week, female/male, n (%)	4 (7.7)	3 (14.3)	7 (13.2)	0.60
Current smoker, n (%)	7 (13.5)	5 (23.8)	5 (9.6)	0.30
Physical activity ≥ 3 times per week, n (%)	21 (42.9)	9 (47.4)	11 (21.2)	0.028*^/^^‡^
Other pain than pain in the feet^§^, n (%)	25 (48.1)	13 (61.9)	41 (77.4)	0.008*
Educational level (high)^‖^, n (%)	17 (32.7)	6 (28.57)	13 (24.53)	0.65
Employed, n (%)	8 (15.4)	6 (28.6)	9 (17.0)	0.38
***Analgesics***				
Paracetamol and NSAIDs, n (%)	11 (21.2)	8 (38.1)	28 (52.8)	0.003*
TCAs, gabapentin, pregabalin, and SNRIs, n (%)	1 (1.9)	2 (9.5)	16 (30.2)	<0.001*
Opioids, n (%)	4 (7.7)	4 (19.1)	11 (20.8)	0.14
***Blood samples***				
HbA1C (mmol/mol), median (IQR)	50.0 (46.0; 55.0)	47.0 (42.5; 57.0)	51.0 (45.5; 61.5)	0.29
Total cholesterol (mmol/L), median (IQR)	3.9 (3.6; 4.4)	4.4 (3.6; 5.8)	3.9 (3.2; 4.5)	0.070
Triglycerides (mmol/L), median (IQR)	1.8 (1.5; 2.9)	2.1 (1.7; 3.2)	2.0 (1.5; 2.9)	0.53
*Neuropathy characteristics*				
NCS abnormal, n (%)	34 (66.7)	12 (57.1)	35 (66.0)	0.13
IENFD abnormal, n (%)	33 (70.2)	20 (95.2)	42 (91.3)	0.01*^/^^†^
Number of IENFD, median (IQR)	2.6 (1.7;4.1)	1.6 (0.7;3.0)	1.2 (0.4;2.7)	0.002*
SFN, n (%)	1 (1.9)	3 (14.3)	2 (3.8)	0.11
LFN, n (%)	6 (11.5)	0 (0)	1 (1.9)	0.064
MFN, n (%)	45 (86.5)	18 (85.7)	50 (94.3)	0.31
*Mental health*				
Quality of life, (NRS 0–10), median (IQR)	8.0 (7.0;9.0)	8.0 (7.0;9.0)	7.0 (5.0;9.0)	0.13
*PROMIS*, *T scores*, *means (SD)*				
Sleep	49.6 (5.8)	52.1 (4.4)	51.2 (4.8)	0.12
Anxiety	49.0 (7.1)	52.6 (6.7)	50.4 (8.8)	0.21
Depression	44.7 (7.2)	51.5 (8.4)	49.0 (9.5)	0.004*^/^^†^
Fatigue	50.4 (8.6)	56.1 (8.6)	56.1 (9.3)	0.002*^/^^†^
*PROMIS*, *number (%) with mild*, *moderate*, *and severe symptoms*				
Sleep	7 (13.5)	5 (23.8)	10 (19.2)	0.50
Anxiety	10 (19.6)	9 (42.9)	17 (32.1)	0.11
Depression	5 (9.8)	8 (38.1)	15 (28.9)	0.010*^/^^†^
Fatigue	21 (40.4)	11 (52.4)	29 (54.7)	0.32
*PROMIS*, *Odds ratio (95% confidence intervals) for having mild*, *moderate or severe symptoms*:				
Sleep	1	2.0 (0.5;7.2)	1.5 (0.5;4.4)	
Anxiety	1	3.1 (1.0;9.3) ^†^	1.9 (0.8;4.8)	
Depression	1	5.7 (1.6;20.3) ^†^	3.7 (1.2;11.1)*	
Fatigue	1	1.6 (0.6;4.5)	1.8 (0.8;3.9)	

*P*-values: between all groups and p < 0.05 for

*pain vs no dysesthesia/pain

^†^dysesthesia vs no dysesthesia/pain

^‡^pain vs dysesthesia.

^§^Other pain than pain in the feet: Pain in at least one other place than the feet (e.g., headache, back/neck pain, and joint pain).

^‖^Educational level (high): Corresponds to a bachelor degree or more. Opioids: at least one of the following: Codeine, methadone, fentanyl patches, tramadol, oxycodone and morphine.

Abbreviations: HbA1C, haemoglobin A1c, BMI; body mass index; NSAIDs, nonsteroidal anti-inflammatory drugs; SNRIs, serotonin noradrenaline reuptake Inhibitors; TCAs, tricyclic antidepressants; NCS, Nerve Conduction Studies; IENFD, Intraepidermal Nerve Fiber Density, SFN, small fiber neuropathy, LFN, large fiber neuropathy, MFN, mixed fiber neuropathy. PROMIS, Patient Reported Outcome Measures.

Missing data: There were fewer than 1% missing values for all variables, except for IENFD with 9.5% missing observations (either because of contraindication or an error in sample shipping and storing), and 4.8% in physical activity.

There was no difference between the groups regarding the proportion of patients with abnormal NCS, whereas almost all patients with dysesthetic or painful DPN had abnormal IENFD values (Table 1).

Patients with dysesthesia and patients with pain had higher scores for depressive symptoms and a higher proportion had abnormal depression scores compared with patients with DPN without dysesthesia/pain. Patients with dysesthesia and pain also had higher fatigue scores than patients without dysesthesia/pain, although there was no difference in the percentages with mild, moderate, or severe interference (Table 1).

Most patients had experienced dysesthesia or pain for 1–5 years and around 40% for more than 5 years as compared to the median time from diabetes diagnosis of around 6 years. The average intensity of pain was higher than the average intensity of dysesthesia (Table 2). Pain interfered more often with activities of daily life than dysesthesia (68% vs 38% with mild to severe interference, *P* = 0.005).

**Table 2:** Duration, intensity, and interference with daily life of pain and dysesthesia.

	Dysesthetic DPN (n = 21)	Painful DPN (n = 53)	*P*
Duration of sensory symptoms in the feet/legs, n (%)			
Equal to or less 1 year	1 (4.8)	7 (13.2)	0.43
More than 1 and up to 5 years	12 (57.1)	24 (45.3)	0.44
More than 5 years	8 (38.1)	22 (41.5)	1.00
Pain/dysesthesia spread from the feet to legs, n (%)	5 (23.8)	24 (45.3)	0.12
Pain/dysesthesia in the hands/arms, n (%)	4 (20.0)	19 (35.9)	0.26
Intensity of pain/dysesthesia the last 24 h, NRS (0–10), mean (SD)	3.5 (2.2)	4.7 (2.8)	0.093
Intensity of pain/dysesthesia the last 7 days, NRS (0–10), mean (SD)	3.8 (1.9)	5.2 (2.4)	0.022
PROMIS interference with daily life, T-scores, mean (SD)	52.1 (7.5)	56.6 (9.2)	0.050
PROMIS interference with daily life (mild, moderate, and severe), n (%)	8 (38.1)	36 (67.9)	0.034
*Mild*	5 (23.8)	16 (30.2)	**-**
*Moderate*	3 (14.3)	18 (34.0)	**-**
*Severe*	0 (0.0)	2 (3.8)	**-**

PROMIS, Patient Reported Outcome Measures. NRS, Numeric Rating Scale.

The groups reported a similar frequency and intensity of symptoms on the NPSI, except for stabbing and pins and needles sensations where patients with painful DPN reported higher intensity compared to dyseshthetic DPN (Table 3).

**Table 3:** Distribution of symptoms using the Neuropathic Pain Symptom Inventory (NPSI).

Symptom description (NPSI)	Dysesthetic DPN (21)		Painful DPN (53)		*P*
	n (%)	*NRS 1–10, mean (SD)	n (%)	*NRS 1–10, mean (SD)	n (%)/mean (SD)
Burning	11 (52.4)	4.3 (1.8)	34 (64.2)	5.0 (2.7)	0.43/0.39
Squeezing	11 (52.4)	4.5 (1.9)	25 (47.2)	5.3 (2.9)	0.80/0.36
Pressure	10 (47.6)	4.2 (1.6)	31 (58.5)	4.7 (2.7)	0.44/0.57
Electric shocks	7 (33.3)	3.1 (2.7)	20 (37.7)	4.8 (2.8)	0.79/0.19
Stabbing	14 (66.7)	3.6 (2.5)	44 (83.0)	5.7 (2.7)	0.21/0.014
Touch-evoked	7 (33.3)	4.0 (2.1)	28 (52.8)	5.5 (2.6)	0.20/0.17
Pressure-evoked	5 (23.8)	3.4 (1.9)	32 (60.4)	4.8 (2.5)	0.009/0.24
Cold-evoked	9 (42.9)	3.0 (1.8)	16 (30.2)	4.4 (2.8)	0.41/0.20
Pins and needles	12 (57.1)	4.0 (2.1)	38 (71.7)	6.3 (2.2)	0.28/0.003
Tingling	13 (61.9)	4.1 (2.4)	36 (67.9)	5.3 (2.9)	0.79/0.19
Sum score (0–100), mean (SD)**		18.3 (16.6)		30.2 (21.1)	0.023

*The NRS (1–10), mean scores are only calculated for those that reported having any of the listed symptoms (more than 0 intensity).

*Mean sum score of all pain/dysesthesia intensities (inclusive those with 0 on the NRS scale).

**Fig 2:**
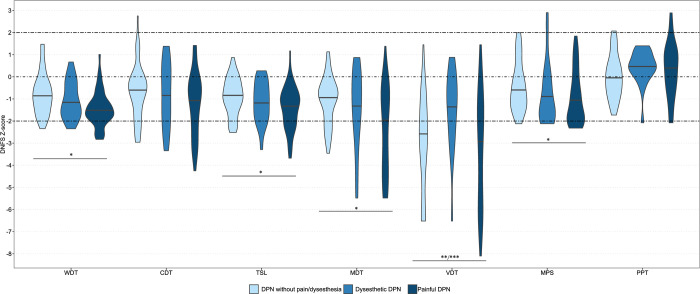
Median of QST Z–scores in DPN without dysesthesia/pain, dysesthetic DPN, and painful DPN. WTD, warm detection threshold; CDT, cold detection threshold; TSL, thermal sensory limen; MDT, mechanical detection threshold; VDT, vibration detection threshold; MPS, mechanical pain sensitivity; PPT, pressure pain threshold. *P* <0.05 is notified by: *pain vs no dysesthesia/pain, **dysesthesia vs no dysesthesia/pain, ***pain vs dysesthesia.

The QST data are summarized in Fig 2 and Table 4 and S1 Fig. The Z-scores for thermal parameters (WDT and TSL) and mechanical parameters (MDT and MPS) were lower in patients with painful DPN than in patients with DPN without dysesthesia/pain, indicating a greater sensory loss of both small and large nerve fiber function in those with painful DPN with a gradient in increased sensory loss from no dysesthesia/pain to dysesthetic and then painful DPN, except for vibration threshold where patients with dysesthetic DPN had lower thresholds compared with the other 2 groups (Fig 2). The QST results showed that only few patients had DMA (8% of patients without dysesthesia/pain and 10% of patients with pain) or increased MPS (2% of patients without dysesthesia/pain and 4% of patients with dysesthesia) (S1 Fig).

**Table 4:** Quantitative sensory testing, median Z–scores.

	DPN without pain (n = 52)	Dysesthetic DPN (n = 21)	Painful DPN (n = 52 (53))	*P*
*Thermal*:				
Warm detection threshold (WDT)	-0.86 (-1.41;-0.30)	-1.24 (-1.76;-0.27)	-1.57 (-1.91;-1.10)	<0.001*
Cold detection threshold (CDT)	-0.59 (-1.60;-0.11)	-0.89 (-1.89;0.14)	-0.92 (-2.20;-0.22)	0.20
Thermal sensory limen (TSL)	-0.87 (-1.38;-0.31)	-1.12 (-1.78;-0.39)	-1.21 (-2.19;-0.81)	0.027*
*Mechanical*:				
Mechanic detection threshold (MDT)	-0.88 (-1.86;-0.40)	-1.35 (-2.30;0.08)	-2.05 (-4.23; -0.71)	0.033*
Vibration detection threshold (VDT)	-2.80 (-4.53;-1.37)	-1.08 (-2.53;-0.33)	-2.53 (-5.53;-1.20)	0.022^†^/^‡^
Mechanical pain sensitivity (MPS)	-0.64(-1.27;0.19)	-1.38 (-1.83;-0.14)	-1.21 (-1.94;-0.04)	0.015*
Pressure pain threshold (PPT)	-0.05 (-0.74;0.64)	0.48 (0.18;1.08)	0.30 (-0.61;1.06)	0.13

All data are medians (IQR). *P*-values are shown between all 3 using Kruskal Wallis test by ranks and if significant, we used Mann-Whitney U test between groups. *P* <0.05 is notified by

*Pain vs no dysesthesia/pain

^†^dysesthesia vs no dysesthesia/pain

^‡^pain vs dysesthesia.

There is 1 missing value in all QST measures except for VDT where there are 11 missing values (5/3/4).

**Fig 3:**
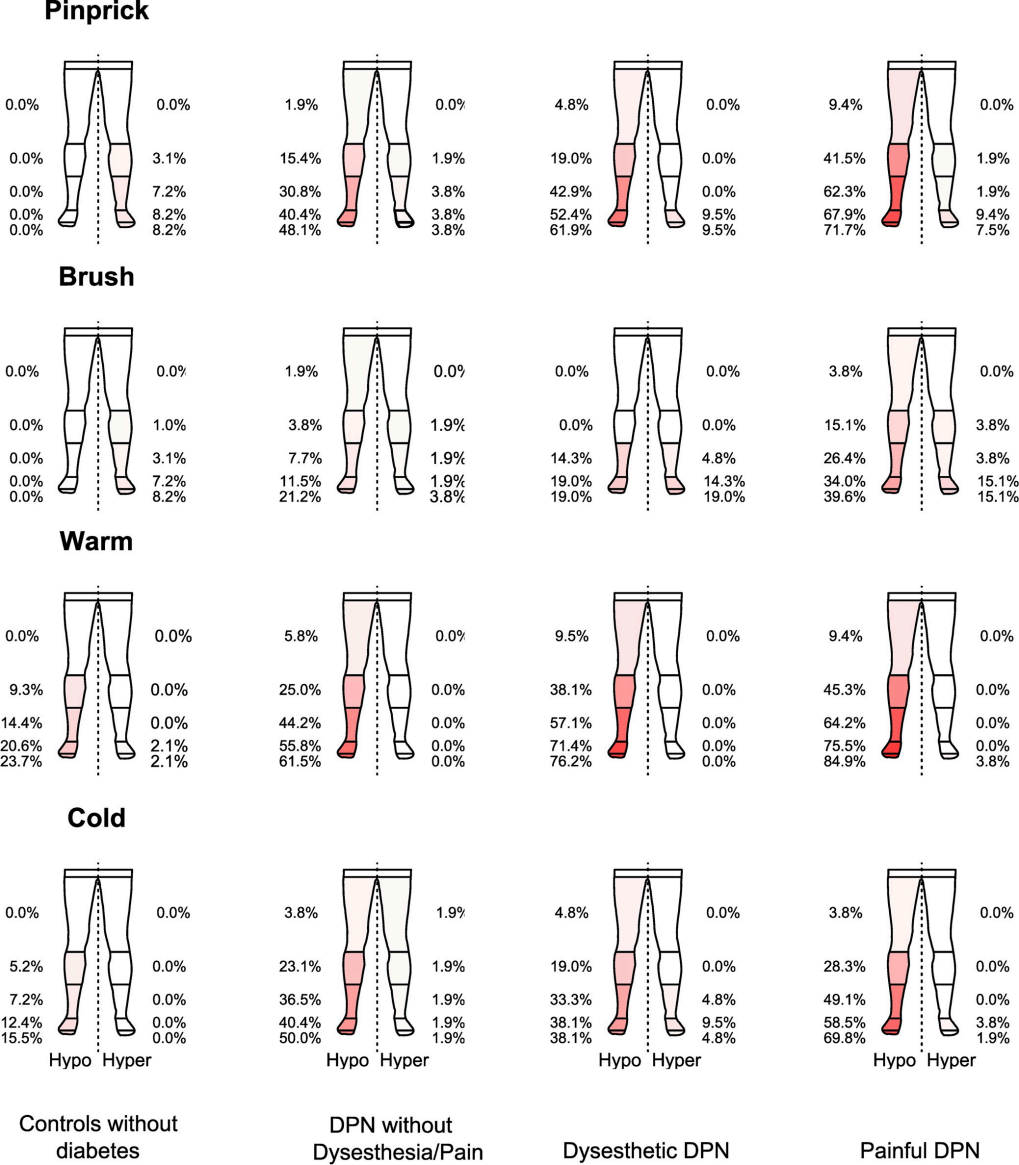
Sensory mapping of lower extremities in controls without diabetes, patients with DPN without dysesthesia/pain, patients with dysesthetic DPN and patients with painful DPN. The correlation between the sum of areas with sensory loss and going from no dysesthesia/pain to dysesthetic DPN and painful DPN. For pinprick: Hypoesthesia: r_s_ = 0.35, *P* = <0.001, Hyperesthesia: r_s_ = 0.13, *P* = 0.16, brush: Hypoesthesia: r_s_ = 0.25, *P* = 0.004, Hyperesthesia: r_s_ = 0.16, *P* = 0.08, warm: Hypoesthesia: r_s s_ = 0.25, *P* = 0.004, Hyperesthesia: r_s_ = 0.14, *P* = 0.12 and cold: Hypoesthesia: r_s_ = 0.17, *P* = 0.06, Hyperesthesia: r_s_ = 0.14, *P* = 0.12. Sensory loss or gain is displayed in percentages from the toes to thighs with hypoesthesia to the left and hyperesthesia to the right. We calculated averages (sum scores) for the right and the left side. There was no difference between sum scores on the left and right leg.

The bedside examination of the lower extremities displayed a length-dependent distribution of sensory loss from the toes to thighs, with an increasing distribution from DPN without dysesthesia/pain to dysesthetic DPN and painful DPN. There was a positive correlation between going from no dysesthesia/pain to dysesthetic DPN and to painful DPN and the increasing number of areas with decreased sensation of pinprick (VF 5.88) (r_s =_ 0.35, *P* <0.001), brush (r_s_ = 0.25, *P* = 0.004) and warmth (40 C°) (r_s_ = 0.25, *P* = 0.004). There was also a positive correlation between the number of areas with hyperesthesia and going from no dysesthesia/pain to painful DPN, but in general weak (Fig 3).

In age and sex matched controls without diabetes, none had hypoesthesia to pinprick and light touch (brush) but a few (8, 2%) had hyperesthesia and 15, 5% had cold and 23, 7% warm hypoesthesia (Fig 3).

## Discussion

In this study, we provided a detailed description of patients with dysesthetic and painful DPN in a group of type 2 diabetes patients with relatively newly diagnosed diabetes. Of the 126 patients with confirmed DPN, 51 had no pain or dysesthesia, 21 had dysesthetic DPN, and 53 had painful DPN. We demonstrated that patients with painful DPN had the largest loss of both small and large sensory fiber function in a symmetric, length-dependent distribution and had a larger distribution of sensory abnormalities. There was also a gradient of increasing sensory loss from DPN without dysesthesia/pain to dysesthetic DPN and to painful DPN. The present findings suggest that dysesthesia is present in an intermediate group with less severe neuropathy than those with pain, supporting our hypothesis.

Recently conducted studies with similar definitions of DPN and painful DPN found younger age and higher levels of HbA1c [7], and higher proportion of females and signs of nephropathy [6, 39] in patients with moderate/severe painful DPN compared to patients without pain. We found a similar tendency for females but not age and HbA1c. Small sample size, shorter duration of diabetes, and type of diabetes could explain some of the discrepancy.

In concert with other studies, we found that patients with painful DPN had more symptoms of depression than patients without pain [7, 40–42]. Patients with dysesthesia also had more symptoms of depression than those with no dysesthesia/pain. In addition, even though they reported less impact on daily life than patients with pain, 38% (24% mild and 14% moderate) reported that dysesthesia had an impact on their daily life, suggesting that dysesthesia, although not labeled as painful by the patients, may be as distressful as pain.

We found that patients with painful DPN were less physically active compared to patients with DPN without dysesthesia/pain and DPN with dysesthesia. A questionnaire study of patients with longstanding type 1 diabetes found that patients who were more physically active reported less neuropathic pain [43]. This could potentially mean that physical activity is protective against pain or that pain reduces physical activity due to pain-related interference with daily life, more depressive symptoms and fatigue.

Only 30% of the patients with painful DPN used medication normally recommended as a first-line therapy for neuropathic pain [44], which is fewer than reported in studies with patients of longer diabetes duration [6, 7, 45] but similar to a study of type 1 and 2 diabetes patients where 37% were treated with at least one of the drugs recommended for neuropathic pain also with longer duration of diabetes [42]. Only 9.5% of patients with dysesthesia received such drugs. It is not known if they requested treatment of their dysesthesia and neuropathic pain drugs have not been tested for dysesthesia despite the impact of dysesthesia. Interestingly, the use of paracetamol and NSAIDs was higher in the pain and dysesthesia groups compared to patients with no pain or dysesthesia. We do not know if that is explained by the fact that they more often had other types of pain or that they are not offered medications recommended for neuropathic pain or if they have stopped using it due to an inadequate effect or side effects [44, 46].

We do not know if dysesthesia presents an intermediate step before development of pain as it is a cross-sectional study and we cannot determine underlying mechanisms from this study. A previous study of patients with neuropathic pain suggested that touch-evoked dysesthesia and pain are transmitted through the same fibers and that the evoked sensation depends on the number of mechanoreceptive fibers that have access to the nociceptive system [47]. Presumable the quality of the symptoms depends to some degree on the type of nerve fiber generating discharges. Based in part on studies from Ochoa and Torebjork using microneurography and ischemic block of nerves in humans it has been suggested that non-painful tingling, pricking and pressing sensations arise from sensory units belonging to large Aβ-fibres, while dull or burning types of pain are generated in C-nociceptive axons [48, 49]. However, it is still discussed how stimuli are encoded to produce the sensation of pain, which may depend on the recruitment of a sufficient number of neurons, activation of specialized high-threshold neurons, or the distribution of activity and interaction across different pathways [50].

The main strength of this study is that the study group consisted of patients diagnosed with confirmed DPN according to widely used standards and patients were recruited from a large patient cohort recruited from both general practitioners and diabetes clinics representative of Danish type 2 diabetes patients with relatively short duration of diabetes [20, 21]. The fact that the clinical examination was carried out at 2 centers could represent a bias, yet we trained the procedures together and checked for consistency regularly throughout the inclusion period. As this was a cross-sectional study, we do not have information on whether patients switch between dysesthesia and pain but this would be important to study in a prospective study. Another limitation is that we did not assess the presence of paresthesia.

In conclusion, there was a gradient in the distribution of sensory loss from DPN without dysesthesia/pain to dysesthesia and then painful DPN, where patients with painful DPN presented with most severe neuropathy. Patients with painful DPN reported less physical activity and patients with pain and dysesthesia had more symptoms of depression than patients with DPN and no pain. The group of patients with dysesthesia resembled patients with pain regarding clinical and demographic data and described their dysesthetic symptoms similar to those with pain according to the NPSI.

## Supporting information

S1 Fig Quantitative sensory testing. Percentages of patients with loss (-%) and gain (+%) of sensory functions in DPN without dysesthesia/pain, dysesthetic DPN, and painful DPN. WTD, warm detection threshold; CDT, cold detection threshold; TSL, thermal sensory limen; MDT, mechanical detection threshold; VDT, vibration detection threshold; DMA, dynamic mechanical allodynia; PHS, paradoxical heat sensation; MPS, mechanical pain sensitivity; PPT, pressure pain threshold. *P* <0.05 is notified by: *pain vs no dysesthesia/pain, **dysesthesia vs no dysesthesia/pain, ***pain vs dysesthesia.
(TIF)

## Abbreviations

CDT: Cold Detection Threshold
DD2: Danish Centre for Strategic Research in Type 2 diabetes
DMA: Dynamic Mechanical Allodynia
DPN: Diabetic polyneuropathy
HbA1c: Glycated hemoglobin
IENFD: Intraepidermal Nerve Fiber Density
LFN: Large fiber neuropathy
MDT: Mechanical Detection threshold
MFN: Mixed fiber neuropathy
MPS: Mechanical Pain Sensitivity
NCS: Nerve Conduction Studies
NPSI: Neuropathic Pain Symptom Inventory
NRS: Numeric Rating Scale
NSAID: Nonsteroidal anti-inflammatory drugs
PROMIS: Patient-Reported Outcomes Measurement Information System
SFN: Small fiber neuropathy
SNRI: Serotonin-Noradrenalin Reuptake Inhibitors
TCA: Tricyclic Antidepressants
TSL: Thermal Sensory Limen
TCNS: Toronto Clinical Neuropathy Scale
QST: Quantitative Sensory Testing
WDT: Warm Detection Threshold
