# Supplementary figures and images for: The characteristics of pain and dysesthesia in patients with diabetic polyneuropathy
Source: PLoS One. 2022 Feb 17;17(2):e0263831. doi: 10.1371/journal.pone.0263831 (PMC8853492; doi:10.1371/journal.pone.0263831)

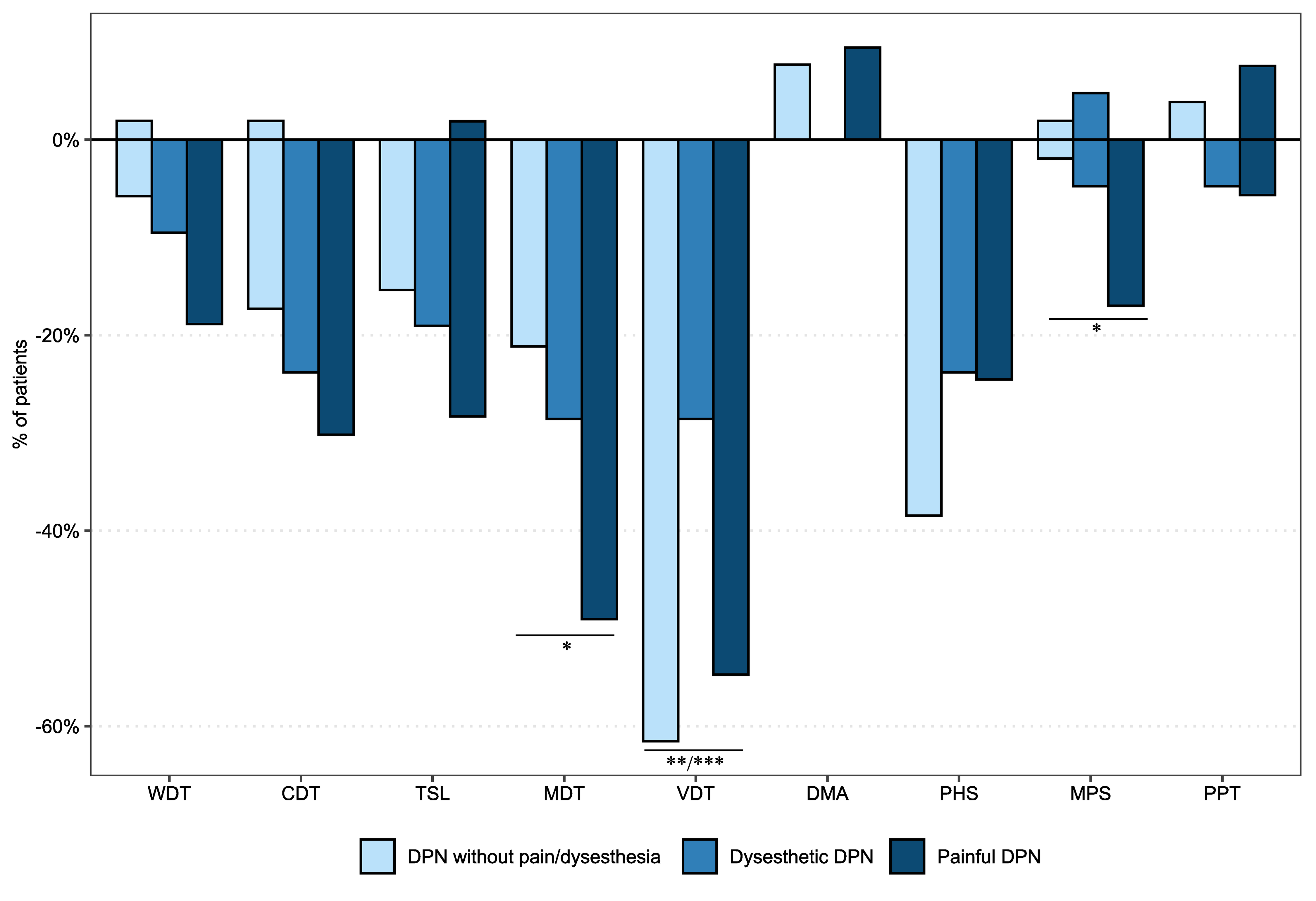

Supplement: S1 Fig — Percentages of patients with loss (-%) and gain (+%) of sensory functions in DPN without dysesthesia/pain, dysesthetic DPN, and painful DPN. WTD, warm detection threshold; CDT, cold detection threshold; TSL, thermal sensory limen; MDT, mechanical detection threshold; VDT, vibration detection threshold; DMA, dynamic mechanical allodynia; PHS, paradoxical heat sensation; MPS, mechanical pain sensitivity; PPT, pressure pain threshold. P <0.05 is notified by: *pain vs no dysesthesia/pain, **dysesthesia vs no dysesthesia/pain, ***pain vs dysesthesia. (TIF) [file pone.0263831.s001.tif]
